# The Deficiency of Tumor Suppressor *Prep1* Accelerates the Onset of *Meis1- Hoxa9* Leukemogenesis

**DOI:** 10.1371/journal.pone.0096711

**Published:** 2014-05-08

**Authors:** Leila Dardaei, Livia Modica, Giorgio Iotti, Francesco Blasi

**Affiliations:** Fondazione Istituto FIRC di Oncologia Molecolare (IFOM), Milan, Italy; II Università di Napoli, Italy

## Abstract

Prep1 and Meis1 ortholog TALE transcription factors have opposing roles in tumorigenesis: *Meis1* serves as an oncogene, Prep1 as a tumor suppressor. We now report that, *Meis1* overexpression in primary *Prep1*-deficient (*Prep1^i/i^*) embryonic hematopoietic cells increases self-renewal potential of cells *in vitro* but not *in vivo*, whereas leukemia is instead obtained when *Meis1* is combined with another oncogene, *HoxA9*. *Prep1^i/i^ Meis1-HoxA9*-generated leukemic cells are less differentiated and grow more aggressively after the second passage in the mouse. These data indicate that *Prep1* represents a barrier to the transforming activity of *Meis1 in vitro*, but its absence is not sufficient to induce early leukemogenesis. On the other hand, the *Prep1^i/i^* background appears to favor the insurgence of mutations that cause a more aggressive *Meis1*-*HoxA9*-generated leukemia. Indeed, the *Prep1^i/i^* leukemic cells upregulate the Polycomb protein Bmi-1 and expectedly down-regulate the *Ink4a/Arf* locus products. Finally, an important feature contributed by the *Prep1^i/i^* background is the post-transcriptional increase in Meis1 protein level.

## Introduction

Prep1 (Pbx-regulating protein 1) and Meis1 (Myeloid ecotropic insertion site 1) are ortholog TALE (three amino acids loop extension) homeodomain transcription factors that competitively interact with the Hox co-factor Pbx (pre-B-cell leukemia homeobox) and with Hox proteins [1], [2], but play opposite roles in tumorigenesis. In primary hematopoietic progenitors, *Meis1* overexpression is unable on its own to transform, but cooperates in the oncogenic activity by accelerating *HoxA9*-induced leukemia [3], [4]. On the other hand, in immortalized mouse embryo fibroblasts, *Meis1* alone is able to transform but only in the absence of *Prep1* [5].

*Prep1* exerts a tumor suppressive function in mice and man [6]. In fact, 40% of the few hypomorphic *Prep1^i/i^* mutant mice (expressing 3–10% of the protein) that survive embryonic lethality [7], develop tumors or pretumoral lesions at late stages in life [6]. In addition, *Prep1* haploinsufficiency (*Prep1^+/−^*) sharply accelerates the death rate of *E*µ*Myc* transgenic mice [8]. Finally, a large percent of human tumors expresses no or reduced Prep1 [6].

Hox homeodomain-containing transcription factors are involved in normal hematopoiesis and leukemogenesis [9], [10], [11], [12]. Their DNA-binding selectivity and specificity is very limited, but is increased by the interaction with Pbx, Meis and Prep cofactors [1], [13], [14]. *Hox* genes promote leukemogenesis by either overexpression or forming chimeric proteins by fusing with other genes [15], [16], [17], [18]. In particular, *HoxA9* is a key regulator of hematopoiesis that has oncogenic functions in leukemogenesis [12]. Its overexpression directly induces leukemia after a long latency [19]. However, *HoxA9* selective collaboration with *Meis1*, but not with *Prep1*, drastically lowers the latency of acute myeloid leukemia (AML) onset in mice [19]. *HOXA9* and *MEIS1* are also expressed in more than 80% of human AML and their expression level is correlated with poor prognosis [20], [21].

Here, we have studied the impact of *Prep1* genotype on *Meis1-HoxA9* induced transformation of hematopoietic cells and subsequent AML induction in mice. We show that, *Meis1* on its own is unable to induce leukemia in primary fetal liver (FL) cells hypomorphic for *Prep1 expression* (*Prep1^i/i^*). However, the absence of *Prep1* favors the *in vitro* immortalization and self-renewal of FL cells induced by *Meis1* or *HoxA9*, whereas *in vivo* it accelerates the rate of appearance of *Meis1-HoxA9*-induced AML but only after two passages in the mouse. As also shown in transformed *Prep1^i/i^* MEFs [5], the *Prep1^i/i^* phenotype leads to increased Meis1 protein level in leukemic cells through a post-transcriptional mechanism. Moreover, *Prep1^i/i^* leukemic cells upregulate Polycomb protein Bmi-1, which results in a decreased expression of Ink4a/Arf cell cycle regulators, p16^Ink4a^ and p19^Arf^, which are known to regulate stem cell potential and proliferation [22]. Overall, the absence of *Prep1* induces a more aggressive leukemia, which may depend on its tumor suppressive function that is based on preventing DNA damage accumulation [23]. Moreover, the less differentiated and highly proliferative phenotypes of *Prep1^i/i^* leukemias may result from the upregulation of Meis1.

## Results

### Meis1 Overexpression in the Absence of Prep1 Induces Serial Transplantation Activity of Mouse Fetal Liver Cells

**Figure 1:**
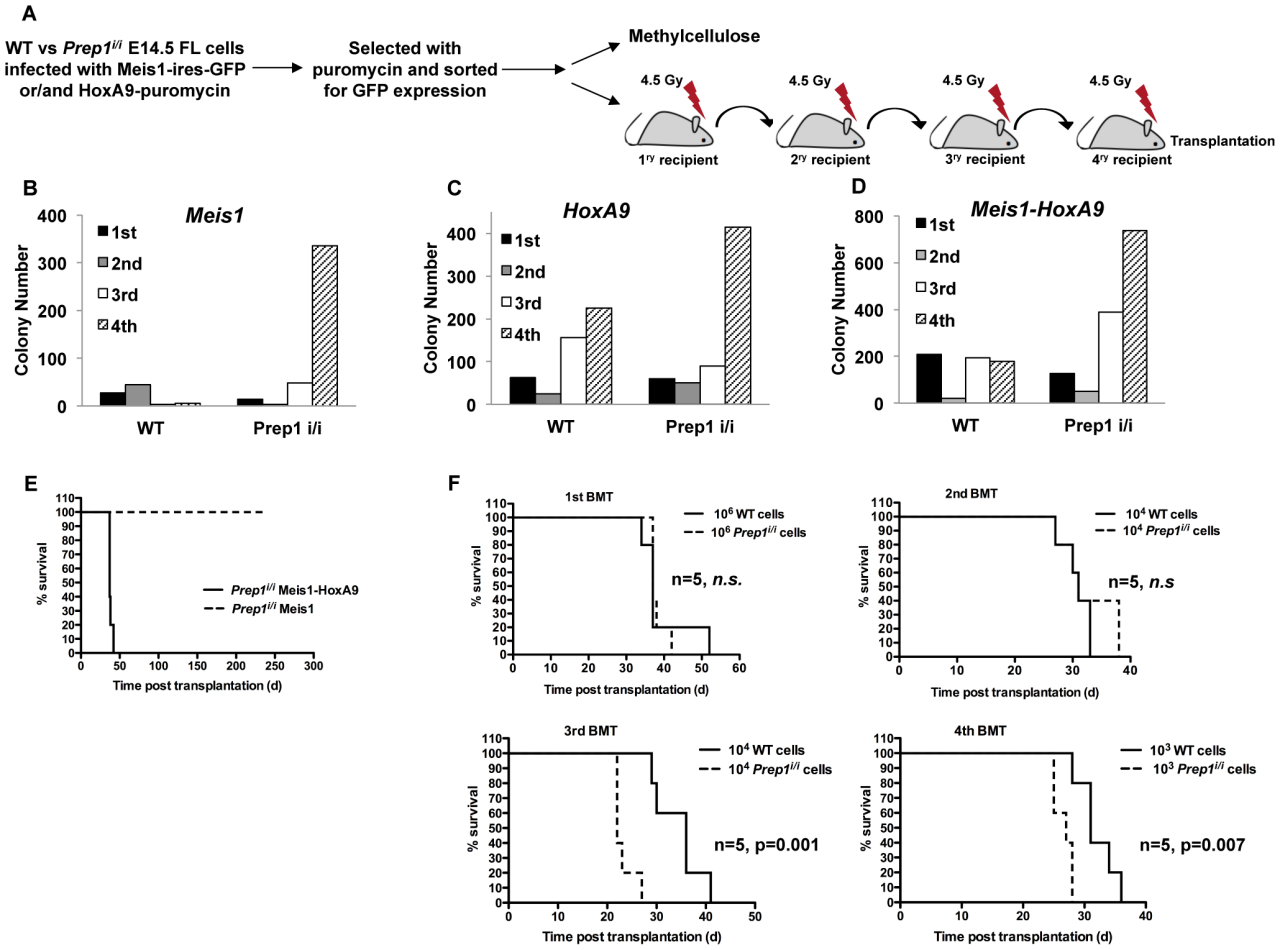
*Prep1* absence accelerates *Meis1-HoxA9* induced myeloid transformation and leukemogenesis. (**A**) Overview of the experimental strategy. FL cells taken from 14.5 WT or *Prep1^i/i^* embryos were transduced with *Meis1* (**B**) or *HoxA9* (**C**) or co-transduced with both oncogenes (**D**) and serially plated in methylcellulose cultures every 7day. Results are shown as colony numbers per 5×10^4^ plated cells for each round of culture. (**E**) Survival curves are shown for cohorts of mice transplanted with 1×10^6^ of *Prep1^i/i^* FL cells transduced with either *Meis1* or *Meis1-HoxA9*. Five mice for each group were used. (**F**) Survival curves are shown for cohorts of mice serially transplanted with the indicated numbers of WT or *Prep1^i/i^* FL cells co-transduced with *Meis1* and *HoxA9*. Five mice per group were used. In the 1st and 2nd Bone marrow transplantation (BMT), the difference between the survival of the mice transplanted with either WT or *Prep1^i/i^* transduced cells was not significant (n.s.). However, in the 3rd and 4th BMTs the p-values of mice survival transplanted with either WT or *Prep1^i/i^* transduced cells were significant (p = 0.001 and p = 0.007, respectively).

To determine whether *Prep1* can exert a tumor suppressive role in leukemia induction, we first compared the transformation rate of *Prep1^i/i^* and WT fetal liver (FL) cells overexpressing the oncogenic *Meis1* **(** **Figure 1A** **)**. E14.5 *Prep1^i/i^* and WT FL cells were retrovirally transduced with *Meis1*-GFP and sorted by FACS (fluorescence-activated cell sorting) for GFP expression. The GFP positive cells were then plated in methylcellulose **(** **Figure 1A** **)**. The ability of FL cells to successfully proliferate and form colonies in methylcellulose upon serial transplantations is normally used to define neoplastic transformation in hematopoietic cells [24]. Serial plating in methylcellulose showed that *Meis1*-overexpression induced self-renewal capacity *in vitro* in *Prep1^i/i^*, but not in WT FL cells **(** **Figure 1B** **)**, an indication of immortalization and neoplastic transformation [24].

When FL cells were transduced with the *HoxA9* oncogene and selected with puromycin, both *Prep1^i/i^* and WT cells were capable of inducing sustained replating, although the efficiency was higher with *Prep1^i/i^* cells **(** **Figure 1C** **)**. When both *Meis1* and *HoxA9* oncogenes were used, both *Prep1^i/i^* and WT cell were capable of inducing sustained replating **(** **Figure 1D** **)** and again the efficiency was higher with *Prep1^i/i^* cells. Thus the absence of *Prep1* is sufficient to induce self-renewal capacity in primary hematopoietic cells. In addition, the absence of *Prep1* appeared to increase the efficacy of both *HoxA9* and of the *Meis1* and *HoxA9* combination.

### The Prep1^i/i^ Mutation Provides a Genetic Background that Causes a More Aggressive Leukemic Phenotype Upon Transformation with Meis1 and HoxA9

Since *Prep1* exerts a tumor suppressive function [6], we tested whether the absence of *Prep1* would allow *Meis1* to induce AML *in vivo* without *HoxA9* overexpression. As shown in **Figure 1E** *Meis1* overexpression in either *Prep1^i/i^* or WT cells was unable to induce AML in mice. Thus the absence of *Prep1* is sufficient to induce *Meis1*-dependent self-renewal *in vitro*, but not AML *in vivo*.

We then tested the role of the *Prep1* genotype on the leukemogenic potential of *Meis1*-*HoxA9* combination. Regardless of the *Prep1^i/i^* or WT genotype, *Meis1-HoxA9*-transduced FL cells induced AML with a similar latency in the primary recipients **(** **Figure 1F** **)**. We then serially transferred the leukemia by transplanting bone marrow (BM) cells. Upon the third bone marrow transplantation (BMT), *Prep1^i/i^* cells promoted AML in recipient mice about 30% earlier than WT cells **(** **Figure 1F** **)** and this property was maintained in subsequent transplantations.

The fact that *Prep1^i/i^* cells induced AML acceleration only after the second transplantation indicates that *Prep1*-deficient cells acquire an aggressive phenotype with the time, probably because of the accumulation of further genetic or epigenetic events. This is in agreement with a primary defect of *Prep1^i/i^* cells to maintain genomic stability [23].

### The Level of Meis1 Protein in Meis1-HoxA9-transduced Cells is Higher in Prep1-deficent than in WT Cells

**Figure 2:**
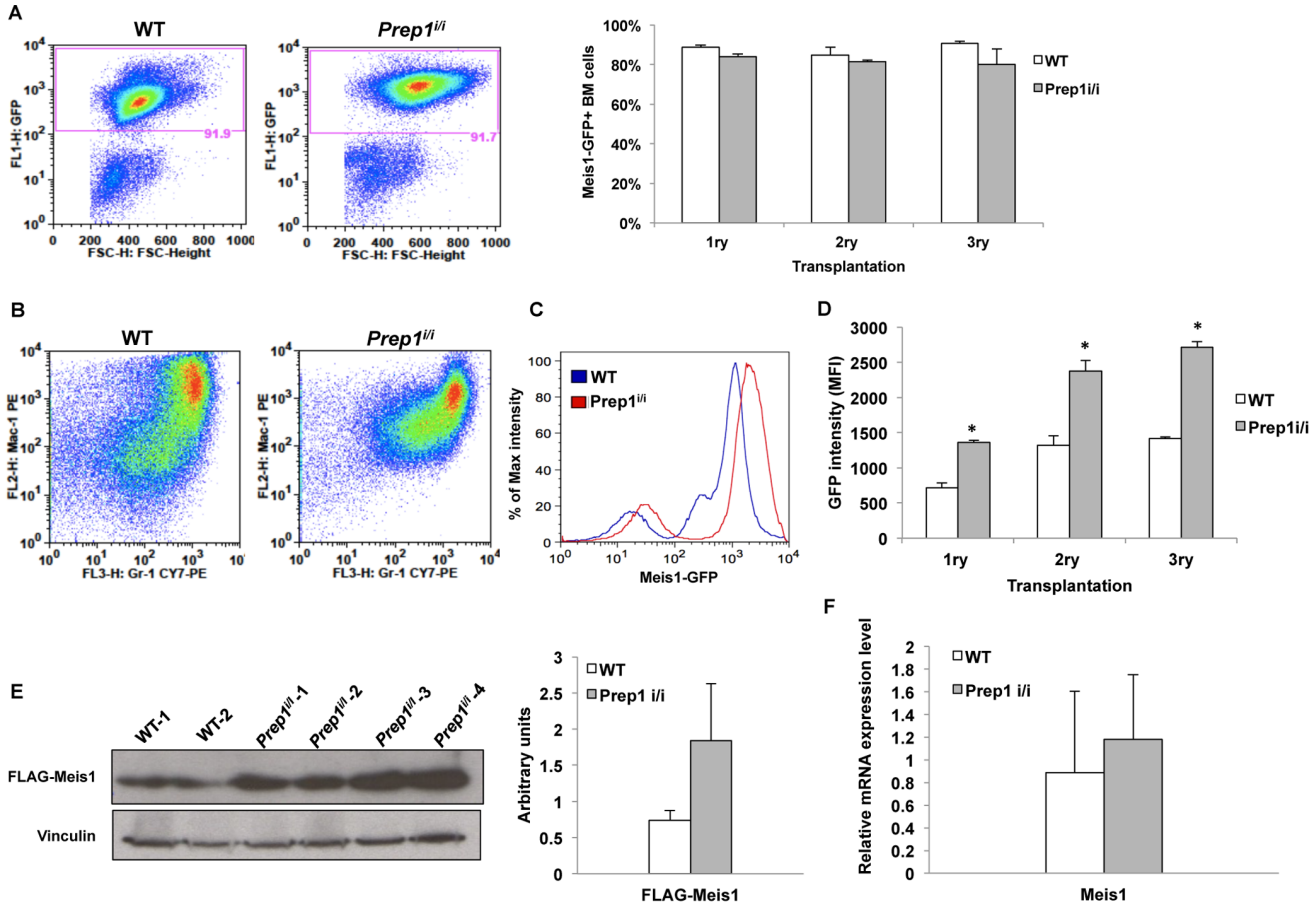
*Meis1* expression is higher in *Prep1^i/i^* leukemic cells compared to WT. (**A**) FACS plot represent the Meis1-GFP-positive cells percent in WT and *Prep1^i/i^* leukemic BM cells (left side). Bar graph shows the summary of Meis1-GFP-positive *Prep1^i/i^* and WT BM cells taken from serially transplanted leukemic mice (right side). Error bars indicate SEM. (**B**) FACS plots represent the Mac-1 and Gr-1 positive cells on GFP-positive cell populations from WT and *Prep1^i/i^* leukemic BM. (**C**) Representative FACS plots of Meis1-GFP expression in *Prep1^i/i^* and WT leukemic BM cells co-transduced with *Meis1*-GFP and *HoxA9*. (**D**) *Meis1*-GFP mean fluorescence intensity of *Prep1^i/i^* and WT BM cells taken from serially transplanted leukemic mice is shown as a graph. *p-value <0.05. Error bars indicate SEM. (**E**) 30 µg of total lysates of leukemic *Prep1^i/i^* and WT BM cells was analyzed by immunoblotting using anti-FLAG antibody. Vinculin was used as loading control. Densitometric analysis was performed using ImageJ64. (**F**) Bar graph shows *Meis1* transcript level measured by real-time PCR in *Prep1^i/i^* and WT leukemic bone marrow cells from serially transplanted mice. Error bars show SD.

*Prep1* overexpression decreases Meis1 half-life in mouse embryonic fibroblasts, and hence reduces its protein level by competing for Pbx1 [5]. To test whether an opposite effect takes place in leukemic cells lacking Prep1, we measured the percent and the signal intensity of GFP-positive cells in *Prep1^i/i^* and WT leukemic BM cells of serially transplanted mice. Since *Meis1* is GFP-tagged the percent of GFP-positive cells and the intensity of the signal represent the frequency and the level of Meis1 in these cells, respectively. The percent of Meis1-GFP positive cells was identical in the two genotypes, indicating equal transduction efficiency in the *Prep1^i/i^* and WT cells **(** **Figure 2A** **)**. However, a clear difference between the two genotypes was observed in terms of *Meis1*-GFP signal intensity **(** **Figure 2C** **)**. Meis1-GFP mean fluorescence intensity, and hence Meis1 protein level, was significantly higher in *Prep1^i/i^* cells compared to WT **(** **Figure 2D** **)**. Moreover, we also checked the FLAG-Meis1 protein level by immuno-blotting, and found it to be higher in the *Prep1^i/i^* leukemic than in WT cells **(** **Figure 2E** **)**. However, the *Meis1* mRNA expression level measured by qRT-PCR did not show any significant difference between *Prep1^i/i^* and WT leukemic cells **(** **Figure 2F** **).** Overall these results suggest that, like in MEFs, the absence of *Prep1* in hematopoietic cells leads to stabilization of Meis1 with subsequent increase of its protein level.

### The Prep1^i/i^ Mutation causes a Less Differentiated and Highly Proliferative Meis1-HoxA9-dependent Leukemias

**Figure 3:**
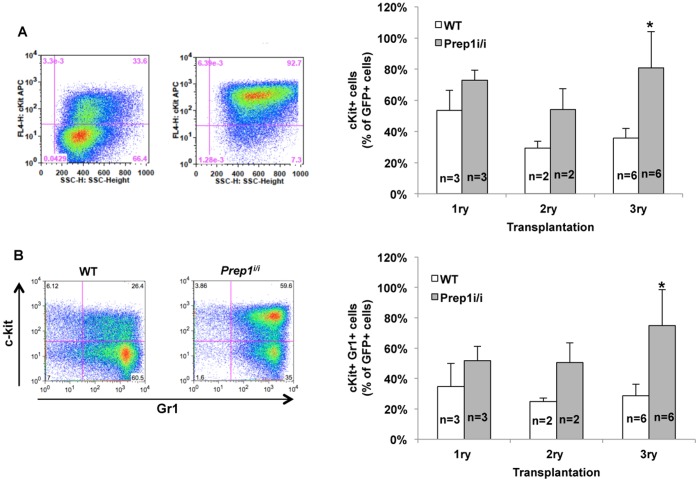
In vitro characterization of *Prep1^i/i^* and WT leukemic cells. (**A**) FACS plots show c-Kit expression on GFP-positive cells in leukemic WT or *Prep1^i/i^* BM cells (right side). Bar graph represents the percent of c-Kit positive cells on GFP-positive population on BM cells from different BMTs (left side). Error bars indicate SD. *p-value <0.001. (**B**) FACS profiles show c-Kit and Gr-1 expressions on BM from mice with AML induced by transplantation of *Prep1^i/i^* or WT cells overexpressing *Meis1-HoxA9* (left side). Bar graph shows the percent of cells co-expressing c-Kit and GR-1 on BM from serially transplanted mice. Error bars represent SD. *p-value <0.001. The number of the mice analyzed for each group is shown on the graph.

Flow cytometric immunophenotyping of the leukemias arisen in two sets of mice transplanted with WT or *Prep1^i/i^ Meis1-HoxA9*-transduced cells, demonstrated that they all were donor-derived as about 90% of the cells expressed GFP **(** **Figure 2A** **)** and displayed Mac-1 and Gr-1 myeloid cell surface antigens **(** **Figure 2B** **)**. However, *Prep1^i/i^* leukemias were less differentiated than WT, exhibiting a higher percent of c-Kit-positive cells **(** **Figure 3A** **)** and a higher frequency of c-Kit and Gr-1 co-expressing cells **(** **Figure 3B** **)**. The less differentiated phenotype of *Prep1^i/i^* leukemic bone marrow cells was not limited to the primary recipient but was constantly observed in secondary and tertiary recipients **(** **Figure 3A and 3B** **)**.

The difference between differentiation states of *Prep1^i/i^* and WT leukemic cells might be both a direct and indirect effect of the absence of Prep1. Indeed, the increased c-Kit expression might be consequent to the higher Meis1 expression induced by the absence of *Prep1*, since *Meis1* has been shown to quantitatively regulate the differentiation arrest of *MLL*-leukemic cells [22].

**Figure 4:**
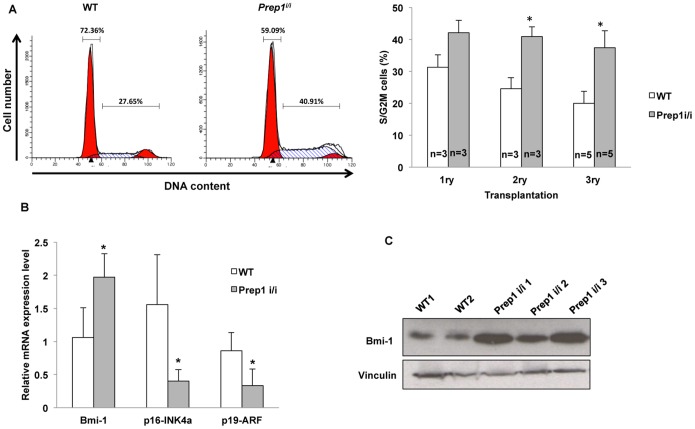
*Prep1*-deficient cells show altered cell cycle activity in *Meis1-HoxA9* leukemia. (**A**) Cell cycle profile of WT or *Prep1^i/i^* leukemic BM cells (right side) performed by flow cytometry. Bar graph summarizes data from cell cycle analysis of leukemic BM cells coming from primary, secondary and tertiary transplanted mice. Error bars indicate the SD. *p-value <0.01. The number of the mice analyzed for each group is shown on the graph. (**B**) mRNA expression level of the indicated transcripts in leukemic BM cells coming from seven mice transplanted with either WT or *Prep1^i/i^ Meis1-HoxA9*-transduced cells. Levels were normalized to the GAPDH expression. Error bars indicate SD. *p-value <0.001 (**C**) Immunoblot represents the Bmi-1 protein level in the total lysate of *Meis1-HoxA9*-transduced leukemic WT or *Prep1^i/i^* BM cells. Vinculin was used as loading control.

A direct comparison of the cell cycle profiles of *Prep1^i/i^* and WT leukemic BM cells induced by *Meis1-HoxA9* showed that more *Prep1^i/i^* leukemic cells accumulated in S/G2/M than WT, whereas their number was reduced in G0–G1 **(** **Figure 4A** **)**. These differences became more evident in the second and third transplantation **(** **Figure 4A** **)**. We have therefore measured the level of various cell cycle regulators in leukemic cells coming from the two genetic backgrounds. Indeed, the higher cycling rate of *Prep1*-deficient leukemic BM cells correlated with a higher expression of the Polycomb group gene *Bmi-1* (both at the mRNA and protein level) **(** **Figure 4B and 4C** **)**, which plays a profound role in cell cycle regulation and maintenance of hematopoietic and leukemic stem cells self-renewal, through transcriptional repression of the *Ink4a/Arf* locus [25]. Indeed, the high level of Bmi-1 in *Prep1^i/i^* leukemic cells correlates with a significant reduction of the p16^Ink4a^ and p19^Arf^ tumor suppressors **(** **Figure 4B** **)**. The impairment of the Bmi-1/Ink4a/Arf axis may explain the less differentiated and highly cycling state of *Prep1^i/i^* leukemic cells. A direct connection between leukemic cells proliferation, *Meis1-HoxA9* and *Bmi-1* has already been established [26].

## Discussion

The *Hox* genes specify cell identity in embryonic development and are involved in several adult processes including hematopoiesis. Likewise, three amino acid loop extension (TALE) proteins, the most important Hox cofactors, are essential in embryonic and adult development including hematopoiesis [12]. For instance, *Pbx1^−/−^* embryos die because of severe hematopoietic defects [27]. *Meis1^−/−^* embryos exhibit profound hemorrhage and lack megakaryocytes [28]. Both *Pbx1* and *Meis1* are required for the maintenance of long-term hematopoietic stem cells [29], [30]. *Prep1* is also implicated in the maintenance of embryonic long-term repopulating hematopoietic stem cells [7] and in adult T cell lymphopoiesis [31].

Consistently, the deregulation of *Hox* and *Meis1* is associated with a number of malignancies including leukemogenesis [12]. However, the *Prep1* function in leukemogenesis is still unexplored. Here we show that in the absence of Prep1 (*Prep1^i/i^*), FL cells are immortalized and transformed by *Meis1* as shown by serial replating assays *in vitro*. Why these cells do not progress to AML in mice remains to be established. In the presence of the *Prep1^i/i^* phenotype, replating efficiency of *Meis1*-transduced cells was as high as with *HoxA9* (**Figure 1B and C**), and the co-overexpression of the two oncogenes had an additive effect **(** **Figure 1D** **)**.

We have previously shown in MEFs that *Prep1* inhibits *Meis1* oncogenicity by destabilizing and hence reducing its protein level [5]. Indeed, the absence of *Prep1* leads to increased *Meis1* protein also in hematopoietic cells, and this level increases in the subsequent transplantations of the leukemic cells **(** **Figure 2** **).** Increased Meis1 is bound to affect the self-renewal of transduced cells, since *Meis1* quantitatively regulates the leukemic stem cell frequency [32], and the aggressiveness of the leukemias, as Meis1 regulates the latency of *MLL* leukemias [22]. Thus it is likely that the progressively higher level of Meis1 in the serial transplantations explains the increased serial replating efficiency, and the increased oncogenic potency.

The absence of *Prep1* has a clear effect on the progression of the *Meis1-HoxA9* dependent AML. *Prep1^i/i^* AML displays a more aggressive phenotype than WT AML only after two passages in the mouse. Also this phenotype therefore can be put in connection with the increased Meis1 level. However, it may also depend on the occurrence of novel mutations. This possibility is in line with *Prep1* being required to maintain genomic stability [23]. Novel mutations would accelerate the onset of the AML after the second bone marrow transplantation (BMT). Regardless of the number of BMTs, the leukemic cells arisen from *Prep1^i/i^ Meis1-HoxA9*-transduced cells are less differentiated and have a higher percent of cycling cells. These phenotypes become more evident with increasing serial transplantations. The absence of *Prep1* might act indirectly to affect the differentiation and proliferation of leukemic cells through the increase of Meis1 **(** **Figure 2** **)**. *Meis1* positively regulates the differentiation arrest and the proliferation potential of leukemic cells [22], [33]. For instance in *MLL*, *Meis1* induces cell proliferation by regulating the expression of *Bmi-1* [22] which encodes a Polycomb group epigenetic repressor protein that regulates the proliferation of normal and leukemic hematopoietic stem cells by silencing cell cycle regulators p16^Ink4a^ and p19^Arf^ [25]. We also find that the highly proliferative state of *Prep1^i/i^* leukemic cells is associated with the increased expression of *Bmi-1* and the subsequent repression p16^Ink4a^ and p19^Arf^ tumor suppressors **(** **Figure 4B and 4C** **)**.

The Bmi-1 repression of *INK4a/Arf* locus may explain the highly cycling status of *Prep1^i/i^* leukemic cells. It remains to be established why the *Prep1^i/i^*-dependent aggressiveness of *Meis1-HoxA9* leukemia appears only at a later stage.

## Materials and Methods

### Mice

*Prep1^i/i^* mice and embryos have been described [7]. Recipient mice (C57Bl6J background, which were CD45.2^+^) were purchased from Charles River and maintained in a specific pathogen free animal facility and housed according to institutional guidelines for a maximum of 1 week before experiments were performed. All animal experiments were performed in accordance with Institutional Animal Care and Use Committee of IFOM and approved by the Italian Ministry of Health (project #110/11). All animal handlings (transplantation, sacrifice etc.,) were in accordance with the guidelines established by EU (directive 2010/63/EU).

Topical anesthesia (ELMA cream) applied to the site 30 minutes prior to intravenous injection of cells. Mice were monitored 3 times per week for three weeks. Starting from 4^th^ week, they were monitored daily. Monitoring for disease was carried out by palpation and observation. Humane endpoints were used to minimize animal suffering when the following distressing signs were observed: 20% weight loss compared to pre-transplantation weight, difficulty to eat, drink, move and enlarged spleen. Mice were euthanized by inhalation of high concentration of CO_2_.

### Plasmids and Retroviral Infection

pMSCVpuro-HA-*HoxA9* was obtained by cloning HA-tagged *HoxA9* cDNA into XhoI/EcoRI restriction sites of pMSCV-puro retroviral vector. MSCV-IRES-GFP carrying FLAG-tagged-*Meis1* was obtained by cloning FLAG-tagged-*Meis1* cDNA into EcoRI restriction site of the vector. Phoenix-Eco (ATCC CRL-3214) cell line was used to produce FLAG-*Meis1-GFP* and HA-*HoxA9* expressing retroviruses. The preparation of single cell suspensions from E14.5 FLs has been described [34]. FL cells were pre-stimulated in 4-cytokine transduction medium (IMDM medium supplemented with 20% heat inactivated fetal bovine serum (FBS), 50 ng/mL SCF, 20 ng/mL IL-6, 10 ng/mL IL-3, and 10 ng/mL FLT31) overnight to induce cell proliferation in order to improve the efficiency of infection. Pre-stimulated FLs were infected with FLAG-*Meis1-GFP*, HA-*HoxA9* or both in retronectin (Takara, 33.3 µg/mL) coated non-tissue culture treated plate (Falcon; 6-well plate polystyrene) in the presence of 4 µg/mL polybrene overnight. 48 h after infection FLAG-*Meis1-GFP* expressing cells were sorted for GFP-positive cells using FACS, and selected with 1 µg/mL puromycin for 3days (for HA-*HoxA9* expression). All cytokines used in this study were purchased from R & D systems (Minneapolis, USA).

### In vivo Transplantation and Leukemogenic Assay

8 to 12 weeks old syngeneic recipient mice were sub-lethally irradiated (4.5 Gy), and indicated numbers of retrovirally transduced FL cells were injected into the tail vein of recipient mice. Indicated numbers of unfractionated bone marrow (BM) cells from primary leukemic mice were serially transplanted into secondary, tertiary, and quaternary recipients. Mice regularly were monitored for signs of leukemia.

### Flow Cytometry Analysis

Leukemic BM cells harvested by flushing femurs and tibias were stained with the following conjugated monoclonal antibodies: c-Kit (2B8 clone), Gr-1 (RB6-8C5 clone), and Mac-1 (M1/70 clone). FLAG-*Meis1-GFP* expression was analyzed by FACS and expression of aforementioned antibodies were assessed on the GFP-positive cell population. Cells co-stained with anti-Mac–1 and anti–Gr-1 were considered as a myeloid lineage. Data were collected using a FACSCalibur cytometer (BD Biosciences, San Jose, CA) and analyzed using FlowJo (Tree Star) software. Antibodies were purchased from eBioscience (San Diego, CA) or BD (BD Pharmingen).

### In vitro Colony Formation Assay

Indicated numbers of *Meis1*, *HoxA9* or *Meis1-HoxA9* transduced FL cells were seeded into 35 mm plates in cytokine-supplemented methylcellulose medium (MethoCult, M3434; STEMCELL Technologies) and incubated at 37°C in 5% CO_2_. Colonies were scored on day 7 of culturing. All colonies were collected from the plate and cells were resuspended, counted and replated (50000 cells/replicate) for a total of 4 platings.

### Cell Cycle Analysis

DNA content analysis was performed by propidium iodide (PI) staining and analyzed by FACS. 1×10^6^ BM cells from leukemic mice were fixed in 75% cold ethanol for 1 h. Cells were then washed twice with PBS plus 1% BSA, and stained in 1 ml PI (50 µg/mL) supplemented with RNase (250 µg/mL) solution for 3 h at room temperature. Data were collected using a FACSCalibur cytometer (BD Biosciences, San Jose, CA) and analyzed using ModFit LT 3.2 software.

### Western Blot Analysis

Western blot analysis was performed following standard procedures. Bone marrow cells were directly lysed in 2X sample buffer and equal amount of the cell lysates were separated in a 10% SDS-PAGE using electrophoresis. The following antibodies were used to detect the proteins of interest: anti-FLAG (clone-M2, Sigma-Aldrich, St-Louis, USA), anti-Bmi-1 (clone-F6, Millipore Massachusetts, USA) and anti-Vinculin (Sigma-Aldrich, St-Louis, USA) antibodies. The ImageJ64 software was used to normalize the results.

### Real-time Quantitative PCR

Total RNA was isolated from *Prep1^i/i^* and WT leukemic BM cells using RNeasy mini kit (QIAGEN, Netherlands) according to the manufacture’s instructions. 500 ng of total RNA was retro-transcribed in buffer containing dN_6_-hexamer random primers, dNTPs, RNase OUT, and SuperScript III reverse transcriptase (Invitrogen, Carlsbad, USA). Primers (Table 1) were ordered from Sigma-Aldrich (St-Louis, USA) and Real-time PCR was performed with SYBR Green I master (Roche, Germany) on a LightCycler 480 system (Roche, Germany). GAPDH was used as control to normalize results. For all experiments, reverse-transcriptase minus controls were performed.

**Table:** **Table1.** List of primers used for Real-time PCR.

Gene	Forward primer	Reverse primer
**GAPDH**	CTCTTCCACCTTCGA	GTCCACCACCCTGTTGCTGTA
**Meis1**	GTTGTCCAAGCCATCACCTT	ATCCACTCGTTCAGGAGGAA
**Bmi-1**	CAACCAGAATCAAGATCACTG	CCATTGGCAGCATCAGCTGAC
**P16^Ink4a^**	GAACTCTTTCGGTCGTACC	CCAGCGTGTCCAGGAAG
**P19^Arf^**	GCTCTGGCTTTCGTGAACATG	TCGAATCTGCACGTAGTTGAG

### Statistical Analyses

The serial transplantation data were analyzed by *GraphPad Prism* software (Version 5.04) to identify significant differences between groups. All other statistical analyses were done by two-tailed Student’s *t* test, and values are expressed as means ± SEM or ± SD. Differences were regarded as significant at p-value <0.05.
